# Ovarian metastasis from thyroid carcinoma: a case report and literature review

**DOI:** 10.1186/s13000-014-0193-9

**Published:** 2014-10-30

**Authors:** Giacomo Corrado, Giulia Pomati, Andrea Russo, Paolo Visca, Cristina Vincenzoni, Lodovico Patrizi, Enrico Vizza

**Affiliations:** Department of Oncological Surgery, Gynecologic Oncology Unit, “Regina Elena” National Cancer Institute, Via Elio Chianesi 53, 00144 Rome, Italy; Surgery Department, Gynecology Section and Obstetrics, Tor Vergata University, Rome, Italy; Pathology Department, “Regina Elena” National Cancer Institute, Rome, Italy; Surgery Department, Gynecologic Oncology Unit, “Regina Elena” National Cancer Institute, Rome, Italy

**Keywords:** Thyroid carcinoma, Ovarian metastasis, Metastatic disease

## Abstract

**Background:**

Papillary thyroid carcinoma is rarely associated with metastatic disease. The most common sites of metastasis are the lungs and bones, while only few cases of ovarian metastasis are described in literature.

**Case:**

We report the case of a 51 years old woman, treated 9 years before for papillary thyroid carcinoma, presenting to our Institute with a pelvic ovarian mass revealed by ultrasound imaging. After bilateral salpingo-oophorectomy, the histologic examination detected a left ovarian metastasis from papillary thyroid carcinoma.

**Conclusion:**

Even if the diagnosis of ovarian metastasis from thyroid carcinoma is often controversial, it should be considered when a woman with an ovarian lesion of unknown origin, has a personal history of malignant thyroid disease.

**Virtual Slides:**

The virtual slide(s) for this article can be found here: http://www.diagnosticpathology.diagnomx.eu/vs/13000_2014_193

## Background

Papillary thyroid carcinoma is the most common histotype of thyroid carcinoma and it is associated to a good prognosis and to a loco regional spread. The presence of distant metastasis is an important prognostic factor, although it is a rare event. Distant metastasis from papillary thyroid carcinoma often occurs decades after the primary tumor and the 70% of patients who die for papillary thyroid carcinoma are disease free after the primary treatment. Moreover, the 30 years mortality rates increase to 43% as a result of a distant recurrence [1,2]. The most common metastatic sites are lung [3] and, following, bone. Instead, rare metastatic sites are brain, parotid, breast, liver, kidney, adrenal glands, ovaries, muscle and skin [4].

Ovaries are the most common metastatic sites from both genital and extragenital primaries, mostly originating in the gastrointestinal tract, and ovarian metastasis represent about 5% to 30% of all ovarian tumors [5,6].

We report a rare case of ovarian metastasis from thyroid carcinoma after 9 years from the diagnosis.

## Case presentation

In December 2013 a 51 years old woman presented to our Gynecologic Oncology Unit, for the presence of a pelvic mass originating from the left ovary, occasionally detected in the ultrasound imaging during a routine check. She was 1 gravida, 1 para, with no previous gynecological pathology in her history. She referred that in 2004, following diagnosis of thyroid carcinoma, she underwent a total thyroidectomy in another hospital. The histological examination revealed a papillary thyroid carcinoma, follicular variant, involving the left thyroid lobe. She received a radio-iodine metabolic adjuvant treatment by administration of 120 mCi ^131^I.

**Figure 1:**
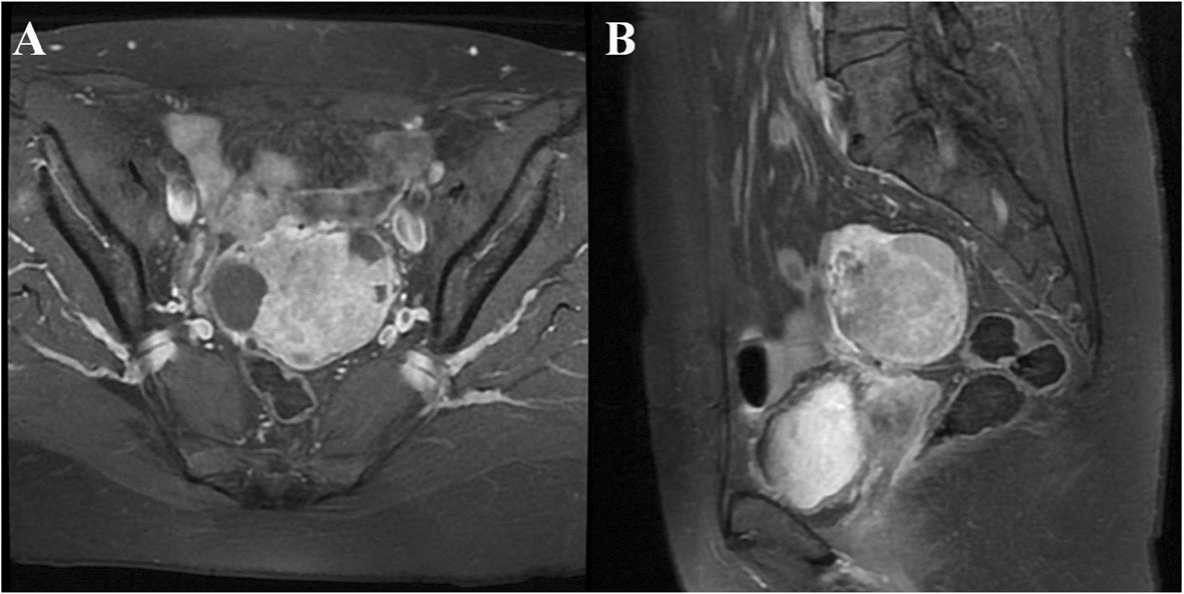
**Pelvic magnetic resonance. A)** Axial fat suppressed T1-weighted image after intravenous gadolinium enhancement. **B)** Sagittal T2-weighted image showing left solid ovarian mass.

**Figure 2:**
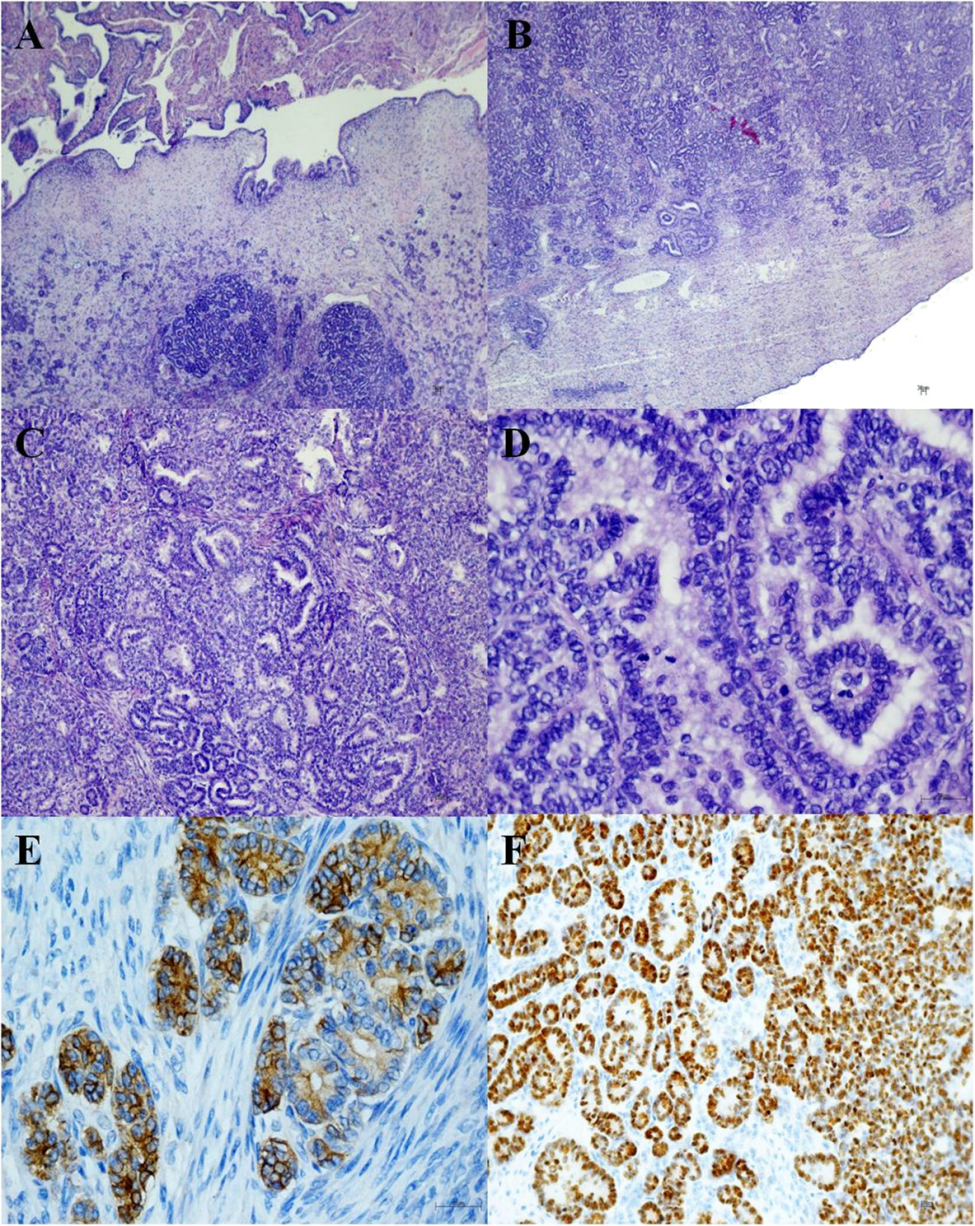
**Microscopic examination and immunohistochemical stains. A)** The ovarian parenchyma is occupied by a thyroid type neoplasm. Note the follicles. (HE 40×). **B)** Massive extension of the neoplasm in the parenchyma. There is no evidence of benign struma ovary or others components of teratoma (HE 40×). **C)** Follicular and papillary components of neoplasm (HE 100×). **D)** Papillary component of neoplasm. Papillae are lined by cells with ground glass nuclei. There are also some mitoses (HE 400×). **E)** Positive reaction of thyroglobulin antibody (HE 400×). **F)** Nuclear positive reaction of TTF-1 antibody (HE 100×).

**Figure 3:**
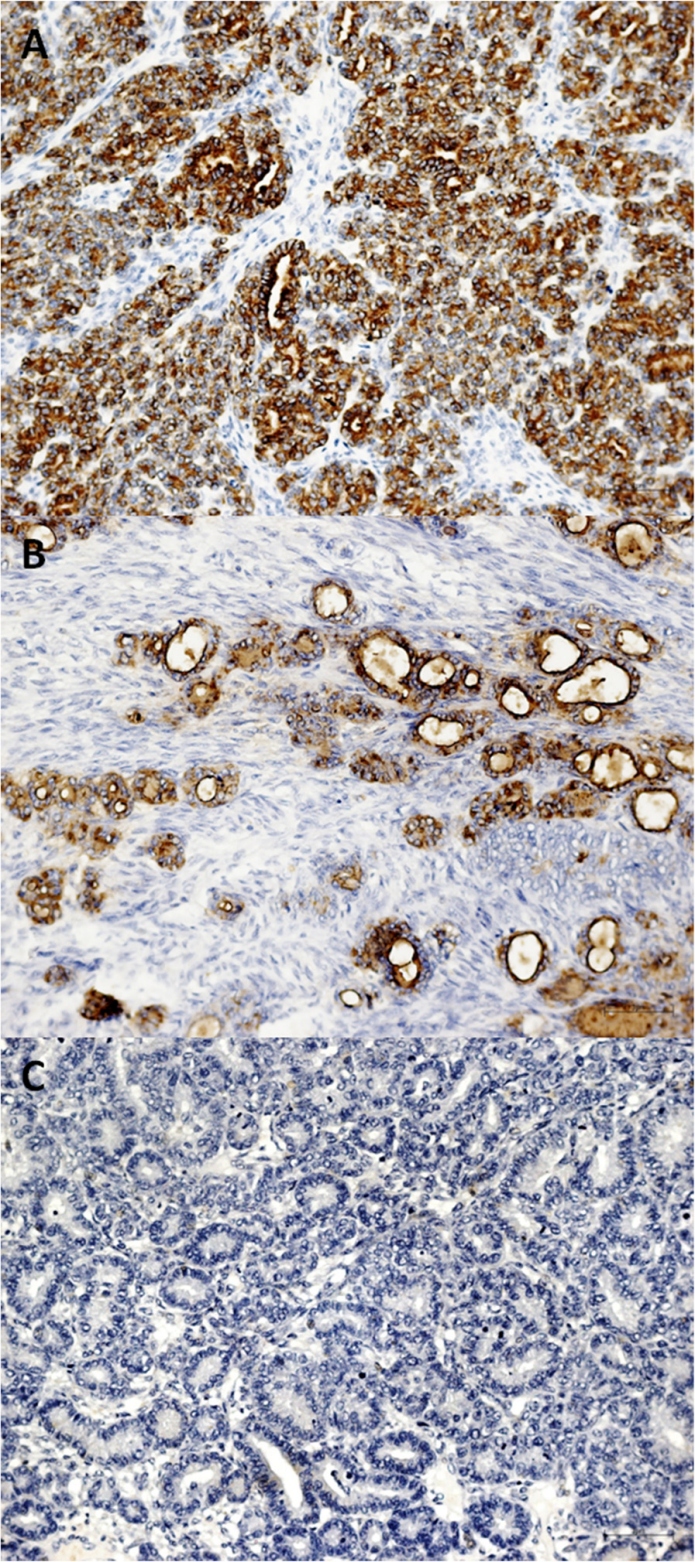
**Other immunohistochemical characteristics. A)** Positive reaction for CK-19 (HE 100×). **B)** Positive reaction for HBME-1 (HE 100×). **C)** Negative reaction for GAL-3 (HE 100×).

No evidence of disease was detected during follow up until December 2013 when an ovarian mass was revealed by ultrasound imaging and at the magnetic resonance it measured 76 × 46 × 62 mm (Figure 1). Normal value resulted for Ca125 and HE4 (respectively 23.9 UI/ML and 100 pmol/L), while thyroglobulin was detectable (0.2 ng/ml). She underwent laparoscopic bilateral salpingo-oophorectomy. The histological exam showed a papillary thyroid carcinoma involving left ovary. The ovarian tumor measured 10 × 5 × 6 cm and weighed 700 g. The section surface was solid and brown. We have performed one sample per centimeter of maximum dimension. Microscopic examination showed that ovarian parenchyma was nearly entirely occupied by thyroid-type neoplasm (Figure 2A-B) characterized by round follicles, of any size, and papillae (Figure 2C). Many follicles were lined by cuboidal, epithelial cells with moderate amounts of cytoplasm and round to oval and ground glass nuclei that exhibited frequent mitotic figures (Figure 2D). These cells were also positive to TTF-1 and Thyroglobulin antibodies (Figure 2E-F). Moreover, Keratin-19 (CK-19) e HBME-1 were positive while Galectin-3 (GAL-3) was negative (Figure 3A-B-C).

This neoplasm was suggestive for a metastasis because there was no evidence of benign struma ovary and the others teratomatous component.

**Figure 4:**
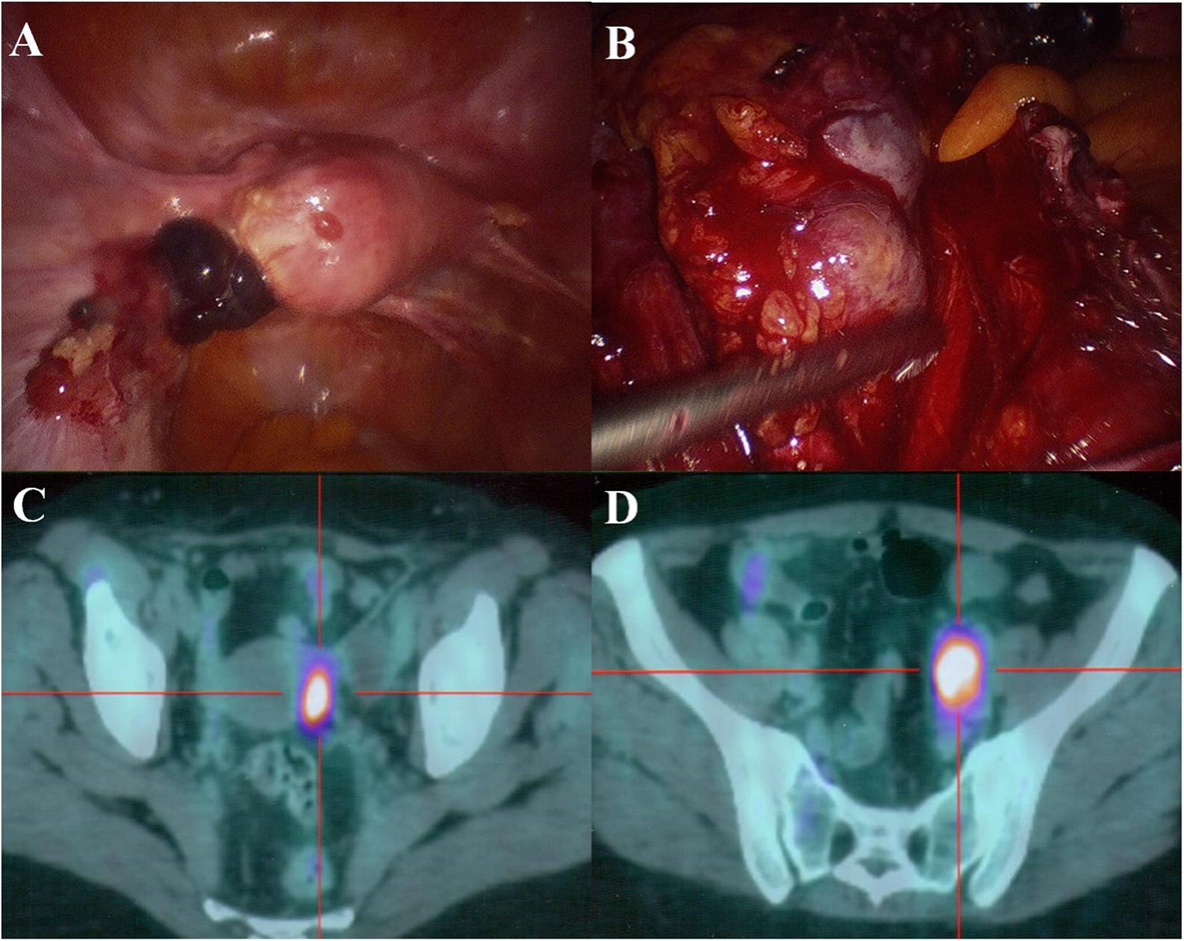
**Laparoscopic and CT/PET characterization of the pelvic recurrence. A)** Laparoscopic image of left pelvic peritoneal carcinomatosis. **B)** The large left pelvic lymphadenopathy. **C)** CT/PET image of a nodular mass near the uterus (SUV 10.6). **D)** Left pelvic lymph nodes (SUV 10.8).

Unfortunately, in March 2014 the CT/PET detected a left pelvic lymph nodes recurrence (SUV 10.8) and a paramedian nodular mass in proximity of the uterus (SUV 10.6). A laparoscopic evaluation showed left pelvic peritoneal carcinomatosis and a large left pelvic adenopathy (Figure 4). A left pelvic lymphadenectomy and a left pelvic peritonectomy were performed. Definitive histological examination showed a metastasis from papillary thyroid carcinoma. Actually, the patient is undergoing biological treatment with multikinase inhibitors.

## Conclusions

Papillary thyroid carcinoma is associated with a good prognosis and with a low metastatic power. A distant metastasis from papillary thyroid carcinoma is a rare event, above all when the recurrence occurs in less common sites. For this reason, rare metastasis is often not considered during the clinical setting.

When an ovarian mass is found to contain cells with features of thyroid carcinoma, a differential diagnoses should have to be considered between thyroid cancer arising from a struma ovarii and ovarian metastasis originating from a primary thyroid carcinoma, since the prognosis and clinical management are different. Thyroid carcinoma originating from a struma ovarii, presenting a papillary histotype in 70% of all cases, is reported to occur much more commonly than an ovarian metastasis from the thyroid. As a matter of fact, struma ovarii are the 5% of ovarian teratomas, 5–10% results in malignant teratomas and metastatic diseases doesn’t reach the 23% of cases. However, when no teratomatous elements and no normal thyroid epithelial tissue are detected in the ovarian lesion, the diagnosis of metastasis with a thyroid origin is suggestive [7]. In our patient, the ovarian parenchyma was completely occupied by thyroid-type neoplasm, there was no evidence of benign struma ovary or others teratomatous component and cells were positive to TTF-1 and Thyroglobulin antibodies.

A review of literature from 1929 to 2013 can confirm the rarity of the ovarian metastasis from thyroid carcinoma. As it is shown in Table 1, only four case reports of ovarian metastasis from thyroid are described in a comprehensive manner [8-11]. The table shows that most of patients affected by thyroid carcinoma were between the fourth and fifth decades of life at the moment of the first diagnosis and underwent I^131^ therapy after primary surgery. Moreover, ovarian metastasis seems to appear more commonly unilaterally. It can be inferred from the description of these cases that well differentiated thyroid carcinomas can give metastasis many years after the primary tumor. As a matter of fact, in Brogioni S et al. [10] case report, the ovarian metastasis occurred almost 5 years after the first pulmonary metastasis and 7 years after the first diagnosis. Also in Pirvu A et al. [11] report, the pulmonary metastasis occurred shortly after the thyroidectomy, while ovarian metastasis 11 years after the first diagnosis of thyroid carcinoma. Furthermore, in the well differentiated thyroid cancer group, papillary histotype seems to give ovarian metastasis more frequently than follicular. In our case ovaries had been the first metastatic site, while in three of the mentioned reports [8,10,11], the ovarian metastasis was associated with a metastatic spread, probably pointing to a biologically more aggressive disease and to a worse prognosis associated to the ovarian involvement. Further 10 cases [12-18] of ovarian spread from thyroid carcinoma are mentioned in literature but, unfortunately, no more details were provided. Besic et al. [12] in his autoptic series reported one case of ovarian metastasis from anaplastic thyroid carcinoma, while Silvesberg et al. [13], always in autoptic series, reported two cases of ovarian metastasis from anaplastic thyroid carcinoma and one from medullary thyroid carcinoma. Others two cases of ovarian metastasis from medullary thyroid carcinoma are only mentioned by Ibanez et al. [14] and Gordon et al. [15]. In another article [17] the ovarian involvement was bilateral. However, in literature there are not enough studies to draw conclusions about prognosis and best clinical management of ovarian metastasis from thyroid cancer. ^131^I scan and serum thyroglobulin are widely employed during the follow up of thyroid cancer and in the assessment of the best therapy to use after surgery, while immunohistochemical stain for thyroglobulin and TTF-1 is often essential in pathologic diagnosis as it has been in our experience. Moreover, Keratin-19 (CK-19) e HBME-1 were positive while Galectin-3 (GAL-3) was negative. This was due to because GAL-3 is a useful marker for diagnosis of low grade thyroid carcinomas [19] while in our case the carcinoma was an high grade.

**Table 1:** **Literature review of ovarian metastasis from thyroid carcinoma**

**Author**	**Year**	**N°**	**Age**	**Primary treatment**	**Histotype**	^**131**^ **I therapy**	**DFS (months)**	**Site of metastasis**	**Surgery of metastasis**	^**131**^ **I therapy**	**Status (months)**
Young RH [8]	1994	1	17	Partial thyroidectomy	Follicular	-	144	Brain, ovaries	Right cystectomy	-	DOD, 150
Logani S [9]	2001	1	34	Total thyroidectomy with lymphadenectomy	Papillary	Yes	132	Ovaries	Left oophorectomy	Yes	NED, 140
Brogioni S [10]	2007	1	38	Total thyroidectomy, with lymphadenectomy	Papillary	Yes	24	Thymus, lungs, ovaries, brain	Bilateral oophorectomy	Yes	DOD, 92
Pirvu A [11]	2013	1	26	Total thyroidectomy with lymphadenectomy	Papillary	Yes	-	Lungs, ovaries	Left ovariectomy	Yes	AWD, 158
Our experience	2014	1	42	Total tyroidectomy	Papillary	Yes	108	Ovaries	Laparoscopic bilateral oophorectomy	No	AWD, 111

NED = no evidence of disease. DOD = death of disease. AWD = alive with disease.

In conclusion, the ovarian involvement by a primary thyroid cancer is a rare event, but it should be considered, since it seems to be a negative prognostic factor worsening the oncological outcome. The histopathologic evaluation, including immunohistochemical stain and the investigation of patient’s history are crucial steps in the diagnosis and clinical management of ovarian metastasis from thyroid cancer.

## Consent

Written informed consent was obtained by patient for publication of this report and any accompanying images. A copy of the written consent is available for review by the Editor-in-Chief of this journal.
